# Physical Warmth and Perceptual Focus: A Replication of IJzerman and Semin (2009)

**DOI:** 10.1371/journal.pone.0112772

**Published:** 2014-11-17

**Authors:** Janneke D. Schilder, Hans IJzerman, Jaap J. A. Denissen

**Affiliations:** 1 Department of Developmental Psychology, Tilburg University, Tilburg, The Netherlands; 2 Department of Social Psychology, Tilburg University, Tilburg, The Netherlands; University of New South Wales, Australia

## Abstract

With the changing of modal research practices in psychology, the grounded cognition perspective (sometimes categorized under the more popular term of “social priming”) has become heavily criticized. Specifically, LeBel and Campbell (2013) reported a failed replication of a study involving what some would call “social priming.” We sought to replicate a study from our own lab (IJzerman & Semin, 2009), to investigate the reproducibility of the reported effect that physical warmth leads to a greater focus on perceptual relations. We also improved our methods to reduce potential experimenter's bias (cf. Doyen, Klein, Pichon, & Cleeremans, 2012). We successfully replicated the finding that a simple cue of physical warmth makes people more likely to adopt a relational focus.

## Introduction

The perspective that cognition is grounded in concrete experiences has been endorsed by most philosophers and researchers throughout human history [1] [2]. Yet, following the cognitive revolution, the dominant metaphor for cognitive functioning became much akin to that of a Turing machine, through the manipulation of arbitrary, amodal symbols [3] [4]. Nevertheless, the past three decades have seen a re-emergence of sensorimotor states in representing cognition (for theoretical perspectives, see [1] [2]; [5]). With the recent ‘scientific revolution’ in psychology however, grounded social cognition findings have become heavily criticized. Most relevantly, LeBel and Campbell [6] reported a failed replication involving what some would coin “social priming”. For these reasons, we replicated a study from our own lab with a much similar topic by IJzerman and Semin ([7]; hereafter IJ&S). The current study has as primary goal to investigate the effect's stability, and as important secondary goal to further minimize experimenter bias, typically associated with this related field of “social priming” [8].

Research on physical warmth revolves around the notion that people's mental representations of relationships are anchored in such experiences. This notion is based on a large array of findings that being in a physically warm (compared to cold) condition leads participants to be more oriented to communal relationships, either behaviorally [9] [10] or cognitively [11] [7] [12] [13] [14] [15]. Importantly, the psychological literature has recently shifted towards robustness and reproducibility of research findings [16] [17] [18]. LeBel and Campbell [6] recently attempted to replicate an effect of semantic “warmth priming”, earlier reported as Study 1 by Vess [19]. Vess found in his Study 1 that participants who reflected on a past romantic breakup (as compared to reflecting on an ordinary event) displayed a greater preference for warm foods, which he took as preliminary support for a relationship between attachment anxiety and warmth cues. However, in their replication study of this effect, LeBel and Campbell [6] were unable to detect the effect.

**Figure 1:**
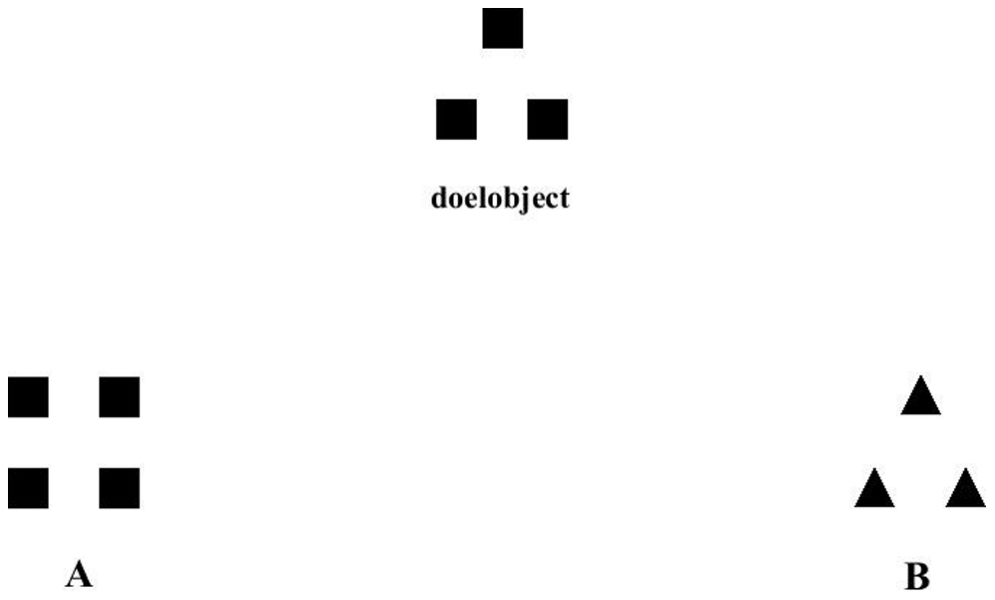
Example of an item used in the perceptual focus task. The target object (in Dutch ‘doelobject’) is a triangle (pattern) consisting of three squares (individual properties). The alternative figure A represents the individual perspective (same individual objects). Alternative figure B represents the relational perspective (same pattern).

We chose to replicate a study of our own lab that deals with a real warm cue, namely IJ&S' Study 3. In this study, participants either sat in a warm or cold room. While doing so, they completed a task that revealed participants' greater preference for relational patterns (vs. individual objects) while in a warm (vs. cold) room (for an example of this task, see Figure 1). Although a number of findings appear to show the robustness of the effect of warmth on social behavior, IJ&S's study enriched this literature by revealing the link between warmth and basic perceptual processes. When we submitted the present replication paper for the first time, the paper was already cited 122 times (according to Google Scholar). However, only one study (i.e., IJ&S *N* = 39) found this effect. Given the present debate on reproducibility and the theoretical implication for embodiment research, we felt this was an important study to replicate.

In IJ&S' study, experimenters were not aware of the study's hypothesis, but in the present study we also ascertained that temperature condition did not influence the experimenter dealing with participants. In so doing, we chose to use mugs filled with either warm or cold fluid which were not touched by the experimenter who was interacting with the participant [10], instead of manipulating room temperature (IJ&S). In this way we intend to add to the present debate on the reproducibility of “warmth priming” and address the issue of the experimenter bias, typically associated with this related field of “social priming” [8].

## Methods

### Participants

A power analysis using G*Power [20], based on IJ&S' effect size.30 indicated that our study required 115 Participants to obtain a power of.8 with an alpha of.05. We conducted our calculations including three independent variables. We measured attachment prior to our manipulations. See also text S1. To account for potential dropout, we recruited 145 participants in Tilburg University's public areas. Participants were approached on weekdays in a two-week time frame in April from 9:30 AM to 4:00 PM. Participants were not approached within 15 minutes before the start of classes and during lunch (12:00–0 PM).

Our sample closely resembles IJ&S' in their Study 3 concerning age and gender (current study: *M*_age_ = 23.15 years, *SD* = 6.78; 48.4% female, IJ&S: *M*_age_ = 21.05 years, *SD* = 3.27; 43.6% female). We excluded 17 participants who either indicated suspicion towards the manipulation 1:0through our debriefing (N = 6), or were non-native Dutch (N = 11; see IJ&S), leaving 128 participants in our final sample.

### Procedure

A second experimenter invited people to participate in a “consumer” study about the paper cups in the University's cafeteria. The guise of the consumer study was used to avoid suspicion towards our temperature manipulation. Before starting the study, participants were requested to sign a consent form. We did not seek approval from the ethics committee since only adults were involved in the study and there was no risk for either emotional or physical health.

A first experimenter filled the mugs and randomly assigned participants to the warm (N = 66) or the cold (N = 62) fluid condition (out of sight for the second experimenter). Then she put the mug in front of the participant without further interacting. The second experimenter, who approached and instructed participants, was thus not aware of the temperature condition. In the case of a participant asking why the cup was filled with water, the second experimenter would state that this would provide a more realistic view for the consumer test (note that only the second, “blind” experimenter interacted with the participant).

The warm or cold water was poured into a paper cup purchased from the University's cafeteria, which allowed the cover story concerning our “consumer test” to be credible. The temperature of the water was preserved in two unmarked thermos flasks filled with water when the faucet was turned to its hottest (or coldest). Temperature was checked at random, using a fluid thermometer (See Table S1 in the Text S1). We refilled the flask when the warm water dropped below 50°C or when above 20°C (cold water), every two hours maximum. The same faucet was used for every refill moment.

The questionnaire programmed in Qualtrics was offered in Dutch and took on average 15 minutes to complete (on a notebook (Acer) or an iPad; the exact questionnaire in Dutch are available via Dataverse). After completing an attachment questionnaire, participants read on their screen that they had to hold the paper cup, but were not allowed to drink from the cup (to avoid any confounding influences). In keeping our cover story credible, participants had to evaluate the attractiveness of the cup by answering three statements (e.g., ‘I find this an attractive mug’; effects were not significant, all *p*s>.33) and one open question (‘What would you like to change about his mug’). After answering these ‘consumer questions’, a perceptual focus task was presented. The perceptual focus task was modeled after Kimchi and Palmer's [21], as used by IJ&S, see Figure 1. We tested participants' suspicion towards the manipulation with one open question at the end. Finally, the experimenter thanked and debriefed participants upon completing the questionnaire.

## Results

Data were analyzed with the use of SPSS 19. In line with IJ&S, we calculated a perceptual focus score, averaging all 24 trials, giving two points for choosing a picture representing a relational focus (“B” in Figure 1) and one for the individual properties (“A”). We replicated IJ&S' statistical analysis (a multiple regression analysis with temperature condition as independent variable, controlling for gender). Language abstraction score was not measured in this study as IJ&S did since we were interested in the association between physical warmth and perceptual focus for the current replication. The analysis confirmed that “warm” participants had a greater relational perspective than “cold” participants, *sr* = .33, *t*(125) = 3.84, *p<*.01, *B* = 0.34. We found similar results without gender as a covariate (Cohen's *d* = .60, *t*(126) = 3.54, *p*<.01). Controlling for gender, the residualized means and standard errors were *M* = 1.69, *SE* = .03, 95% CI [1.63, 1.76] for the ‘warm’ participants and *M* = 1.51, *SE* = .03, 95% CI [1.44; 1.58] for the ‘cold’ participants. In the original IJ&S study, residualized means and standard errors (controlling for gender) were, *M* = 1.69, *SE* = .03, 95% CI [1.64, 1.75] for the ‘warm’ participants and *M* = 1.61, *SE* = .02, 95% CI [1.56, 1.66], for the ‘cold’ participants. Our findings thus replicate IJ&S' findings that a simple cue of warmth makes people more likely to adopt a relational perspective. A post-hoc power analysis indicated that the power of the current study was.79, approximating typically recommended standards [22]. Data of this study are publically available.

## Discussion

We replicated a study earlier reported by IJzerman and Semin [7] and found a comparable effect. Our finding provides empirical support for the notion that “warm” (as compared to “cold”) participants focus more on relational (vs. individual) properties. It is true that our replication was not “exact”, but no study in psychology can be so. Replications fall on a range from close to conceptual, and our study can be found more towards the anchor of being a “close replication” [17]. We thus acknowledge small changes in method (room vs. cup; lab vs. public space; Utrecht vs. Tilburg University), but we feel that we offered an improved method, further confirming IJ&S' effect, and, because of our larger sample, more accurately estimated the effect size. A limitation is that neither study included a neutral condition (e.g., a mug with water that has room temperature). This way, it is not possible to determine whether differences are due to the effect of the cold mug (temperature decrease), the warm mug (temperature increase), or both. Future studies might address this issue.

The current findings further add to the present debate on the reproducibility of “warmth priming” and the issue of the experimenter bias, typically associated with this related field of “social priming” [8]. As a closing comment, we highly encourage researchers from independent labs to further replicate these social embodiment findings, as replicability is an ever-increasing important facet of psychological research.

## Supporting Information

Text S1 **The inclusion of the variable attachment in our research and outcomes are discussed here.** Table S1. Temperature of the water was checked at random and was listed in this table.
(DOCX)

Data S1 (SAV)
