# Correction: Physical Warmth and Perceptual Focus: A Replication of IJzerman and Semin (2009)

**DOI:** 10.1371/journal.pone.0129636

**Published:** 2015-05-28

**Authors:** Janneke D. Schilder, Hans IJzerman, Jaap J. A. Denissen

There is an error in the first sentence of the fourth paragraph in the Procedure section of the Methods. The data are available from Open Science Framework instead of Dataverse. The correct sentence is: The questionnaire programmed in Qualtrics was offered in Dutch and took on average 15 minutes to complete (on a notebook (Acer) or an iPad; the exact questionnaire in Dutch are available via https://osf.io/ijxhr/).
