# Supplementary material for: Physical Warmth and Perceptual Focus: A Replication of IJzerman and Semin (2009)
Source: PLoS One. 2014 Nov 17;9(11):e112772. doi: 10.1371/journal.pone.0112772 (PMC4234632; doi:10.1371/journal.pone.0112772)
Supplement: Text S1 — The inclusion of the variable attachment in our research and outcomes are discussed here. Table S1. Temperature of the water was checked at random and was listed in this table. (DOCX) [file pone.0112772.s001.docx]

Supplement

Text S1.The role of attachment on the association between physical warmth and perceptual focus

We sought to extend the effect by examining an interaction effect with a relevant individual difference variable, attachment [1]. We measured attachment style before our temperature manipulation through Fraley, Waller, and Brennan's [2] Revised Experiences in Close Relationships (ECR-R) self-report measure of romantic attachment anxiety (model of self) and avoidance (model of others) to explore potential interaction effects of attachment and temperature onto perceptual focus.
 Neither Anxiety (sr=.04, t(124) = .48, B = .27, p= .63) nor Avoidance (sr = .03, t(124) = .33, B= .20, p = .74) predicted Perceptual Focus as main effect and no significant interaction effects were obtained for Anxiety X temperature condition (sr = -.04 , t(124) = -.50, β = -.04, p= .62) and for Avoidance X temperature condition (sr = .06 , t(124) = .72, β = .06, p = .48).
 There are a number of reasons why we could not detect the effect of attachment on the association between physical warmth and perceptual focus. First, it is possible that no moderation exist, and that the effects of temperature are due to other factors than being relationship relevant [3]. Second, it could well be that the present set of questions on attachment security do not accurately cover the relevant aspects of attachment for social thermoregulation, which is likely rooted in resource regulation [4] [5]. Finally, it is also fathomable that the lack of effect can be explained by most (M_Secure_ = 5.25, SD_Secure_ = .62) of our participants being securely attached (for scoring, see Fraley et al.’s guidelines [2]).

Table S1. Temperature in degree Celsius of the warm and cold water measured at random moments in minutes after filling the thermos flask

| Minutes after refill | Warm | Cold |
| --- | --- | --- |
|  |  |  |
| 06 | 58 | 16 |
| 03 | 59 | 15 |
| 05 | 56 | 17 |
| 114 | 50 | 18 |
| 45 | 54 | 18 |
| 116 | 51 | 18 |
| 07 | 58 | 16 |
| 36 | 57 | 15 |
| 111 | 52 | 18 |
| 07 | 58 | 17 |
| M Temperature | 55,3 | 16,8 |
|  |  |  |

References Supplement
1 IJzerman H, Karremans JC, Thomsen L, & Schubert TW (2013) Caring for Sharing.
 Social Psychology 44: 160-166.

2 Fraley RC, Waller NG, & Brennan KA (2000) An item response theory analysis of self-
 report measures of adult attachment. Journal of personality and social psychology 78:
 350.

3 Stevens JC (1982) Temperature can sharpen tactile acuity. Perception & psychophysics 31:
 577-580.

4 Beckes L & Coan JA (2011) Social baseline theory: The role of social proximity in
 emotion and economy of action. Social and Personality Psychology Compass 5: 976-
 988.

5 Beckes L, IJzerman H, & Tops M (2014) Toward a Radically Embodied Neuroscience of
 Attachment and Relationships?. Available at SSRN 2429522.
